# *IKZF1* deletion is associated with a poor outcome in pediatric B-cell precursor acute lymphoblastic leukemia in Japan

**DOI:** 10.1002/cam4.87

**Published:** 2013-05-09

**Authors:** Daisuke Asai, Toshihiko Imamura, So-ichi Suenobu, Akiko Saito, Daiichiro Hasegawa, Takao Deguchi, Yoshiko Hashii, Kimikazu Matsumoto, Hirohide Kawasaki, Hiroki Hori, Akihiro Iguchi, Yoshiyuki Kosaka, Koji Kato, Keizo Horibe, Keiko Yumura-Yagi, Junichi Hara, Megumi Oda, for the Japan Association of Childhood Leukemia Study (JACLS)

**Affiliations:** 1 Department of Pediatrics, Kyoto Prefectural University of Medicine, Graduate School of Medical ScienceKyoto, Japan; 2 Department of Pediatrics, Oita UniversityOita, Japan; 3 Clinical Research Center, National Hospital Organization Nagoya Medical CenterNagoya, Japan; 4 Department of hematology/oncology, Hyogo Prefectural Children's HospitalKobe, Japan; 5 Department of Pediatrics, Mie UniversityTsu, Japan; 6 Department of Pediatrics, Osaka UniversitySuita, Japan; 7 Department of Hematology Oncology, Children's Medical Center, Japanese Red Cross Nagoya First HospitalNagoya, Japan; 8 Department of Pediatrics Kansai, Medical UniversityHirakata, Japan; 9 Department of Pediatrics, Hokkaido UniversitySapporo, Japan; 10 Department of Pediatrics, Osaka General Medical CenterOsaka, Japan; 11 Department of Pediatrics, Osaka City General HospitalOsaka, Japan; 12 Department of Pediatrics, Okayama UniversityOkayama, Japan

**Keywords:** Acute lymphoblastic leukemia, *CRLF2*, *IKZF1* deletion, pediatric

## Abstract

Genetic alterations of *Ikaros family zinc finger protein 1* (*IKZF1*), point mutations in *Janus kinase 2* (*JAK2*), and overexpression of *cytokine receptor-like factor 2* (*CRLF2*) were recently reported to be associated with poor outcomes in pediatric B-cell precursor (BCP)-ALL. Herein, we conducted genetic analyses of *IKZF1* deletion, point mutation of *JAK2* exon 16, 17, and 21, *CRLF2* expression, the presence of *P2RY8-CRLF2* fusion and F232C mutation in *CRLF2* in 202 pediatric BCP-ALL patients newly diagnosed and registered in Japan Childhood Leukemia Study ALL02 protocol to find out if alterations in these genes are determinants of poor outcome. All patients showed good response to initial prednisolone (PSL) treatment. Ph^+^, infantile, and Down syndrome–associated ALL were excluded. Deletion of *IKZF1* occurred in 19/202 patients (9.4%) and *CRLF2* overexpression occurred in 16/107 (15.0%), which are similar to previous reports. Patients with *IKZF1* deletion had reduced event-free survival (EFS) and overall survival (OS) compared to those in patients without *IKZF1* deletion (5-year EFS, 62.7% vs. 88.8%, 5-year OS, 71.8% vs. 90.2%). Our data also showed significantly inferior 5-year EFS (48.6% vs. 84.7%, log rank *P* = 0.0003) and 5-year OS (62.3% vs. 85.4%, log rank *P* = 0.009) in NCI-HR patients (*n* = 97). *JAK2* mutations and *P2RY8*-*CRLF2* fusion were rarely detected. *IKZF1* deletion was identified as adverse prognostic factor even in pediatric BCP-ALL in NCI-HR showing good response to PSL.

## Introduction

Acute lymphoblastic leukemia (ALL) is the most common pediatric malignancy and is an important cause of morbidity and mortality in children [1, 2]. Despite progress in therapy, approximately 20% of pediatric patients with B-cell precursor (BCP)-ALL with no adverse prognostic factors still experience relapse [3–5]. Recent genome-wide profiling studies of pediatric ALL identified a number of novel genetic alterations that target key cellular pathways for lymphoid growth and differentiation and that are associated with treatment outcome [6]. Using high-resolution single-nucleotide polymorphism (SNP) arrays and genomic DNA sequencing, Mullighan et al. [7–10] and other groups revealed that alterations in genes encoding transcriptional regulators of B-lymphocyte development and differentiation, including *PAX5*, *EBF1*, and *IKZF1*, were observed in approximately 40% of patients with BCP-ALL. They also found that deletion of *IKZF1* was very frequent in *BCR-ABL*–positive ALL and in the lymphoid blast crisis of chronic myeloid leukemia [11]. Importantly, they also revealed that *IKZF1* deletion or mutation was associated with a very poor outcome, even in *BCR-ABL*–negative BCP-ALL [12–14]. The prognostic impact of *IKZF1* deletion was also confirmed by several other groups studying pediatric BCP-ALL [15–17]. Thus, *IKZF1* deletion has recently been considered as a prognostic marker for pediatric BCP-ALL and might be useful for risk stratification [16]. In addition, activating point mutations of *JAK2* coexisted with *IKZF1* deletion in pediatric BCP-ALL with a BCR-ABL-like gene expression signature and a very poor outcome [18]. Other studies reveal that overexpression of *cytokine receptor–like factor 2* (*CRLF2*) due to *IgH@-CRLF2* fusion resulting from immunoglobulin heavy-chain locus (*IgH*@) translocation or *P2RY8-CRLF2* fusion resulting from the interstitial deletion of the pseudoautosomal region 1 (PAR1) of either of the sex chromosomes (Xp22/Yp11) was significantly associated with *JAK2* mutations, *IKZF1* alterations, and a poor outcome in BCP-ALL [19–25]. Furthermore, Hertzberg et al. [21] demonstrated that patients with Down syndrome–associated ALL harbored *JAK2* mutations in association with altered *CRLF2* overexpression, which in some patients was caused by an activating somatic mutation, F232C, in the *CRLF2* gene. In this study, we sought to test whether deletion of *IKZF1*, dysregulation of *CRLF2, JAK2* mutations, or deletions in *PAX5 or EBF1* are prognostic determinants in Japanese pediatric BCP-ALL patients.

## Materials and Methods

### Patient cohort and samples

From April 2002 to May 2008, 1139 patients aged 1–18 years with newly diagnosed BCP-ALL (standard risk, SR = 457, high risk, HR = 543, and extremely high risk, ER = 139) (risk factors for classification are described in Table S#1) were enrolled in the JACLS ALL study and assigned to three risk-stratified ALL02 protocols [26, 27]. The diagnosis of BCP-ALL was based on morphological findings on bone marrow aspirates and immunophenotype analyses of leukemic cells by flow cytometry. Conventional cytogenetic analyses using G-banding method were done as part of the routine workup. Molecular studies using quantitative RT-PCR for the detection of *BCR-ABL*, *ETV6-RUNX1*, *MLL-AF4*, *MLL-ENL*, *MLL-AF9,* and *TCF3*(*E2A)-PBX1* were performed as part of the routine workup. None of the cases of *BCR-ABL*-positive ALL and infant ALL was included. Down syndrome–associated ALL was excluded from the genetic analysis. Thus, 542 patients were included in this study. As the study was multi-institutional and the registry of patients was drawn from 93 hospitals, appropriate DNA/RNA specimens at diagnosis could be obtained from only 202 of the 542 patients in this group. A comparison of the clinical characteristics of patients with and without DNA/RNA specimens is shown in Table 1. There was no difference between these two cohorts in age at diagnosis, gender, NCI risk [28], and other variables except for initial WBC count. Informed consent was obtained from the patients' guardians according to the Declaration of Helsinki and the protocols of treatment, and the genetic study were approved by the institutional review boards of the participating institutes.

**Table 1:** Comparison of characteristics in 543 high-risk BCP-ALL patients depending on whether they were included in the genetic analyses

Number of patients	202	340	*P-*value
	Analyzed	Nonanalyzed	
Gender (male/female)	106/96	186/154	0.66
Age (yrs) at diagnosis, median (range)	5 (1–18)	6 (1–17)	0.24
WBC count (cells/μL), median (range)	21,350 (1300–400,800)	12,635 (430–26,500)	<0.01
NCI risk group, SR/HR	108/94	189/151	0.63
*ETV6-RUNX1*/hyperdiploid			0.85
Yes	69	113	
No	133	227	
SCT in 1st CR (*n*)	0	9	0.03
Observation period, median (range)	5.4 (0.5–8.9)	5.1 (0.1–9.0)	0.47

WBC, white blood cell; NCI, National Cancer Institute; SR, standard risk; HR, high risk; SCT, stem cell transplantation; CR, complete remission.

### Determination of abnormalities in *IKZF1* and other genes by multiplex ligation-dependent probe amplification

Genomic DNA was isolated from diagnostic bone marrow or peripheral blood samples using the Qiagen DNeasy Blood and Tissue kit according to the manufacturer's instructions (Qiagen, Venio, the Netherlands). DNA was analyzed using the SALSA multiplex ligation-dependent probe amplification (MLPA) kit P335-A4 according to the manufacturer's instructions (MRC Holland, Amsterdam, the Netherlands). This kit includes probes for *IKZF1*, *CDKN2A*, *CDKN2B*, *PAX5*, *ETV6*, *RB1*, *BTG1*, *EBF1,* and the PAR1 region, which includes *CRLF2, CSF2RA*, and *IL3RA*. PCR fragments generated with the MLPA kit were separated by capillary electrophoresis on an ABI Prism 3130 Genetic Analyzer (Applied Biosystems, Foster City, CA). Peak area was measured using GeneMapper software (Applied Biosystems). The relative copy number, obtained after normalization against controls, was used to determine genomic copy number of each gene as follows: 0 (<0.6, i.e., biallelic loss), 1 (0.6–1.47, i.e., monoallelic loss), 2 (1.47–2.6, i.e., normal copy number), or 3 or more (>2.6, i.e., gain) [29].

### Detection of *CRLF2* overexpression by real-time RT-PCR

Total RNA was extracted from diagnostic bone marrow or peripheral blood samples using the RNeasy Mini Kit (Qiagen) according to manufacturer's instructions. cDNA was synthesized using the SuperScript First-Strand Synthesis System (Invitrogen, Carlsbad, CA) according to manufacturer's instructions. Real-time RT-PCR was conducted using the 7300 Real-Time PCR System (Applied Biosystems) with SYBR Green II (Takara Bio, Tokyo, Japan). Relative expression of target mRNA was determined using the comparative threshold (ΔC_T_) method, in which the C_T_ value of the glyceraldehyde-3-phosphate dehydrogenase (GAPDH) internal control mRNA is subtracted from that of the target mRNA. Data are expressed as the ratio of target mRNA to GAPDH mRNA (calculated as 2^ΔCT^). The primer pairs used in this study are listed in Table S2. Overexpression of *CRLF2* was defined as expression tenfold or greater than the median expression value based on a previous report [23]. A total of 107 specimens were available for the *CRLF2* study.

### Detection of *P2RY8-CRLF2* fusion by RT-PCR

The *P2RY8-CRLF2* fusion was detected by RT-PCR or MLPA kit P335-A4 in 202 patients. The primers used are listed in Table S2.

### *CRLF2* mutation analysis

Using cDNA samples with altered *CRLF2* expression, the presence of the *CRLF2* F232C point mutation was detected by direct sequencing. The primers used are listed in Table S2. Appropriate RNA samples were available from all the 16 patients who overexpressed *CRLF2*.

### *JAK2* mutations analysis

Genomic DNA was extracted from diagnostic bone marrow or peripheral blood samples of patients harboring an *IKZF1* deletion. Primers were used to amplify exons 16, 20, and 21 of *JAK2* (accession number NM 004972). The PCR product was analyzed by direct sequencing using a BigDye Terminator sequencing kit (Applied Biosystems). The primers used are listed in Table S2. Appropriate DNA samples from all patients with the *IKZF1* deletion were available for the *JAK2* mutations study.

### Statistical analysis

Estimation of event-free survival (EFS) and overall survival (OS) was performed using the Kaplan–Meier method and the differences were compared using the log rank test. A *P*-value <0.05 (two sided) was considered significant. EFS and OS were defined as the times from diagnosis to event (any death, relapse, secondary malignancy, or failure of therapy) and from diagnosis to death from any cause or to the last follow-up. Patients without an event of interest were censored at the date of last contact. The median follow-up times for EFS and OS were 5.22 and 5.34 years, respectively. Hazard ratios for probability of relapse between subgroups were calculated using univariate Cox models. Multivariate analysis was performed using a Cox regression model, which was adjusted for other risk factors: age at diagnosis and initial WBC count. Other comparisons were performed using the chi-square test or Fisher exact test, as appropriate.

## Results

### Frequency of *IKZF1* deletion, *CRLF2* overexpression, and *JAK2* mutations in patients in the JACLS ALL02 HR cohort

Deletion of the *IKZF1* gene was identified in 19/202 patients (9.4%) and *CRLF2* overexpression was noted in 16/107 patients (15.0%). The *P2RY8-CRLF2* fusion and *CRLF2* F232C mutation were very rare (1/202 and 0/16 patients, respectively). A recent study demonstrated that gain of *CRLF2* copy number was observed in BCP-ALL with overexpression of *CRLF2* [24]. In consistent with this report, 8 (50.0%) of 16 patients with altered *CRLF2* expression harbored gain of *CRLF2* copy number in our cohort (8/16 vs. 2/91, *P* < 0.01). In addition, no *JAK2* mutations (exons 16, 20, 21) were detected.

### Association of clinical and genetic features with *IKZF1* deletion and *CRLF2* overexpression

The comparison of patient characteristics depending on *IKZF1* deletion and *CRLF2* overexpression is summarized in Table 2. Gender, initial WBC count, and NCI risk did not differ between patients with these genetic variations. *IKZF1* deletion was more frequently observed in older patients (1–9 years vs. 10–18 years, *P* = 0.008). In terms of concurrent chromosomal/genetic abnormalities, *IKZF1* deletion was observed in 1/68 of *ETV6-RUNX1*–positive + hyperdiploid/trisomy 4, 10, and 17 (triple trisomy [30]) patients compared to 18/134 of patients without those abnormalities (*P* = 0.0059). These findings suggest that *IKZF1* deletion and chromosomal abnormalities with good prognosis are mutually exclusive. Similarly, the status of *CRLF2* overexpression did not significantly correlate with gender, age at diagnosis, initial WBC count, or NCI risk. However, *CRLF2* overexpression was significantly associated with chromosomal abnormalities with an incidence of normal karyotype + hyperdiploid/triple trisomy of 12/36 compared to 4/71 for other karyotypes (*P* = 0.014). However, in our cohort, only one *P2RY8-CRLF2* fusion and no *CRLF2* F232C mutations were detected. In addition, this *P2RY8-CRLF2*–positive case did not have *IKZF1* deletion. *JAK2* mutations in exons 16, 20, or 21 were not detected in the 19 patients with *IKZF1* deletion. These findings suggest that genetic events in association with *IKZF1* deletion in our cohort might be different from those previously reported [21, 24, 25].

**Table 2:** Association of clinical and genetic features with *IKZF1* deletion and *CRLF2* overexpression

	*IKZF1* deletion			*CRLF2* OE		
				
	Yes	No	*P-*value	Yes	No	*P-*value
Total	19	183		16	91	
Gender			0.51			0.38
Male	8	98		11	47	
Female	11	85		5	44	
Age (yrs) at diagnosis						
Median	10	5	0.08	4.0	5.0	0.41
1–9	9	143	0.009	14	63	0.16
10–18	10	40		2	28	
WBC count (×10^3^cells/μL)						
Median	23,430	20,810	0.68	22,000	24,240	0.87
<100	17	165	0.92	14	78	0.31
≥100	2	18		2	13	
NCI risk group			0.15			0.14
SR	7	101		10	41	
HR	12	82		6	50	
Karyotype			0.003			0.014
No fusion genes	16	101		16	50	
(normal karyotype)	(7)	(32)		(5)	(18)	
(hyperdiploid/ triple trisomy)*	(0)	(28)		(7)	(6)	
(others)**	(8)	(39)		(1)	(25)	
(undetermined)	(1)	(2)		(3)	(1)	
Fusion genes	3	82		0	41	
(*ETV6-RUNX1*)	(1)	(40)		(0)	(20)	
(*TCF3(E2A)-PBX1*)	(2)	(37)		(0)	(18)	
(11q23)	(0)	(5)		(0)	(3)	

* Triple trisomy indicates trisomy 4, 10, and 17.

** Karyotype other than normal karyotype, hyperdiploid, triple trisomy, and 11q23 abnormality, showing a negative result in screening for chimeric fusions, as described in the Materials and Methods.

OE, overexpression; NCI, National Cancer Institute; SR, standard risk; HR, high risk.

### IKZF1 deletion, not CRLF2 overexpression, was strongly associated with a poor outcome

**Figure 1:**
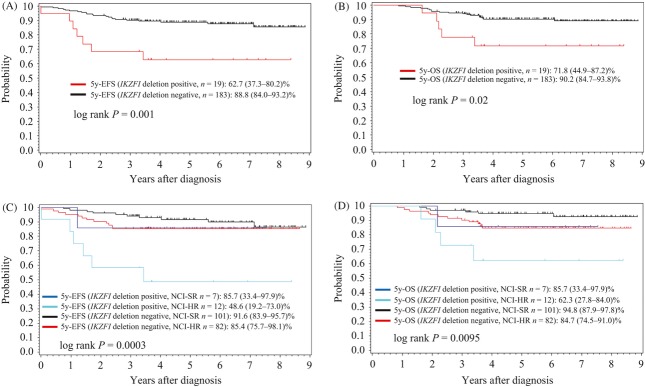
Kaplan–Meier estimates of event-free survival (EFS) and overall survival (OS) in BCP-ALL patients enrolled in the JACLS ALL02 HR cohort (*n* = 202). (A) EFS and (B) OS for patients with or without *IKZF1* deletion in this cohort. (C) EFS and (D) OS for patients with or without *IKZF1* deletion according to NCI risk group.

As seen in Figure 1, the 5-year EFS and OS for the patients with *IKZF1* deletion were inferior to those without *IKZF1* deletion. To compare our data internationally, we assigned patients in our cohort into NCI high risk (NCI-HR) and NCI standard risk (NCI-SR). In NCI-HR group (*n* = 94), the 5-year EFS and OS for patients with *IKZF1* deletion (*n* = 12) was inferior to those without *IKZF1* deletion. However, in NCI-SR patients (*n* = 108), the 5-year EFS and OS for the patients with *IKZF1* deletion (*n* = 7) was comparable to those without *IKZF1* deletion (Fig. 1). As summarized in Table 3, univariate analysis revealed that *IKZF1* deletion was associated with a significantly inferior EFS (*P* < 0.01). *IKZF1* deletion was also associated with a significantly inferior EFS by multivariate analysis (*P* = 0.03, Table 4). However, NCI risk alone did not affect the EFS by univariate (*P* = 0.06) in our cohort. These findings suggest that *IKZF1* deletion is an independent prognostic factor in pediatric patients with BCP-ALL in JACLS-HR and NCI-HR groups in this cohort.

**Table 3:** Univariate cox model of event-free and overall survival of analyzed patients

Variable	Hazard ratio	*P*	95% CI
Event-free survival			
Age (yrs) at diagnosis (1–9 vs. 10–18)	2.923	<0.01	1.404–6.089
Gender (male vs. female)	1.279	0.51	0.611–2.679
WBC count (×1000 cells/μL)(≥100 vs. <100)	2.682	0.03	1.090–6.600
NCI risk (HR vs. SR)	2.067	0.06	0.975–4.381
*IKZF1* deletion (Yes vs. No)	3.701	<0.01	1.579–8.675
Overall survival			
Age (yrs) at diagnosis (1–9 vs. 10–18)	3.188	<0.01	1.406–6.089
Gender (male vs. female)	0.819	0.63	0.361–1.857
WBC count (×1000 cells/μL)(≥100 vs. <100)	2.678	0.05	0.994–7.216
NCI risk (HR vs. SR)	2.787	0.02	1.146–6.776
*IKZF1* deletion (Yes vs. No)	3.069	0.03	1.139–8.269

NCI, National Cancer Institute; SR, standard risk; HR, high risk.

**Table 4:** Multivariate cox model of event-free and overall survival of analyzed patients

Variable	Hazard ratio	*P*	95% CI
Event-free survival			
Age (yrs) at diagnosis (1–9 vs. 10–18)	2.586	0.02	1.192–5.610
WBC count (×1000 cells/μL)(≥100 vs. <100)	2.882	0.02	1.163–7.138
*IKZF1* deletion (Yes vs. No)	2.668	0.03	1.086–6.553
Overall survival			
Age (yrs) at diagnosis (1–9 vs. 10–18)	3.016	0.01	1.281–7.102
WBC count (×1000 cells/μL)(≥100 vs. <100)	2.866	0.04	1.049–7.829
*IKZF1* deletion (Yes vs. No)	2.049	0.18	0.723–5.807

On the contrary, 5-year EFS achieved in the patients with *CRLF2* overexpression was 100%, suggesting *CRLF2* overexpression was not adverse prognostic factor in our current cohort.

### Other microdeletions were not associated with a poor outcome

In our cohort, the frequencies of deletions of other genes tested were 81/202 (40%) for *CDKN2A,* 69/202 (34%) for *CDKN2B*, 59/202 (29%) for *PAX5*, 47/202 (23%) for *ETV6*, 12/202 (5.9%) for *RB1*, 15/202 (7.4%) for *BTG1*, and 22/202 (11%) for *EBF1*. None of these genetic alterations were associated with altered probability of relapse regardless of the presence/absence of *IKZF1* deletion (Table S3).

## Discussion

To the best of our knowledge, this is the first report of *IKZF1, CRLF2,* and *JAK2* alterations in pediatric BCP-ALL in Japan. The frequency of *IKZF1* deletion in JACLS ALL02 HR patients was 19/202 (9.4%), which is consistent with previous reports [7, 11, 17]. In this study, univariate and multivariate analyses revealed that the *IKZF1* deletion was an independent factor for inferior EFS in pediatric BCP-ALL patients. More specifically, *IKZF1* deletion was strongly associated with a poor outcome in the NCI-HR patient group. As NCI-HR is defined by age and initial WBC alone, it consisted of patients who showed a good response to initial prednisolone administration, which corresponds to JACLS-HR, and those who did not, which corresponds to JACLS-ER. Nevertheless, the fact that *IKZF1* deletion affects outcomes in the NCI-HR group in our cohort indicates that this mutation is an independent prognostic factor irrespective of the initial prednisolone response. Thus, we believe that early risk stratification in pediatric BCP-ALL patients should be based on a new risk stratification system including *IKZF1* status [16].

In terms of prognostic value of *CRLF2* alteration, our study demonstrated that *CRLF2* overexpression was not necessarily associated with a poor prognosis as well as did the previous study [25]. The contradictory results reported by Cario et al. [24], in which *CRLF2* overexpression was associated with poor EFS in the ALL-BFM 2000 protocol (6-year EFS 28% vs. 71%, *P* = 0.001), were thought to be mainly due to the effect of the *P2RY8-CRLF2* fusion, and not simply *CRLF2* overexpression. The frequency of *CRLF2* overexpression was 16/107 (15.0%) in this cohort, which is comparable to previous reports. However, we could not confirm that the activating mutation of *JAK2* and *CRLF2* overexpression caused by *P2RY8-CRLF2* rearrangement was associated with *IKZF1* alteration. These findings might explain that *CRLF2* overexpression was not associated with a poor prognosis in the current cohort.

In previous reports, excluding patients with Down syndrome, the incidences of point mutations of *JAK2* exons 16, 20, and 21 causing gain of function have been associated with *IKZF1* deletion and *P2RY8-CRLF2* fusion: 87.5% of patients with *JAK2* mutations had *IKZF1* alterations (*P* = 0.001) [18] and 30–100% of patients with *JAK2* mutations had *P2RY8-CRLF2* fusion [21–25]. On the other hand, we detected only one patient with *P2RY8-CRLF2* fusion of 202, and this patient did not have point mutations of *JAK2* exon 16, 20, or 21 in this analysis. Although the reason for the discrepancy between our data and that of others is not clear, there might be several ones to be in consideration. First, the frequency of genetic alterations might depend on the ethnicity. It is interesting that genetic alterations of *JAK* and *CRLF2* are relatively frequent in Hispanic patients [23]. Although there is no report of the analysis of *JAK2* and *P2RY8-CRLF2* fusion in pediatric BCP-ALL from other Asian countries, it might be possible that the frequency of *JAK2* and *P2RY8-CRLF2* fusions are relatively low among Asian patients. Second, our genetic analysis carried out in this study was not comprehensive. For example, we were not able to analyze the *IgH@-CRLF2* fusion due to lack of the materials for FISH analysis and the type of *CRLF2* genomic aberrations might be varied in studied cohorts [23–25, 31, 32]. We did not analyze *JAK1* or *JAK3* which may also play roles in leukemogenesis, although the frequency of the alterations in these genes is thought to be considerably lower than those in *JAK2* [18]. Third, it is also possible that JACLS ALL02 HR cohort might not include BCP-ALL cases with *BCR-ABL* like gene expression signature, in which *JAK2* activating mutation and *CRLF2* genomic aberration are concurrently present with *IKZF1* deletion. Thus, we are planning to carry out the genetic analysis of the BCP-ALL cases treated according to JACLS ALL02 ER group which might include more BCP-ALL cases with *BCR-ABL* like gene expression signature.

In conclusion, *IKZF1* deletion should be a valuable prognostic marker to include in future algorithms for early risk stratification in the treatment of pediatric BCP-ALL. Further studies are required to clarify the genetic alterations instead of *P2RY8-CRLF2* fusion and/or *JAK2* mutations, which may cooperate with *IKZF1* deletion in these leukemic patients in Japan.

## Supporting Information

**Table S1.** Risk stratification in JACLS ALL02 trial.

**Table S2.** Primer list used in this study.

**Table S3.** Impact of other genetic alterations on outcome depending on *IKZF1* deletion.
