# Supplementary material for: IKZF1 deletion is associated with a poor outcome in pediatric B-cell precursor acute lymphoblastic leukemia in Japan
Source: Cancer Med. 2013 May 9;2(3):412–9. doi: 10.1002/cam4.87 (PMC3699852; doi:10.1002/cam4.87)
Supplement: Supplementary file 1 [file cam40002-0412-SD1.docx]

Table S1. Risk stratification in JACLS ALL02 trial

| Risk | Definition |
| --- | --- |
| Standard risk (SR) | PGR and meet all of the following criteria  (1) initial WBC <10,000 /μl, (2) age at diagnosis 1-9 yrs, (3) not CNS3 |
|  |  |
| High risk (HR) | PGR and meet one of the following criteria;  (1) initial WBC>10,000 /μl, (2) age at diagnosis >10 yrs  (3) t(1;19), (4) 11q23 (excluding t(4;11) leukemia), (5) CNS3 |
|  |  |
| Extremely high risk (ER) | All of PPR,  Mixed-lineage/AUL and t(4;11) leukemia irrespective of PSL response |

Abbreviations: PGR, prednisolone (PSL) good responder (blast counts in peripheral blood are less than 1,000 / μl after one week PSL treatment); PPR, PSL poor responder; CNS, central nervous system.

Table S2. Primer list used in the current study

| *JAK2* | Exon 16 | Forward | GTCAGCTCCCATCCAGAAAC |
| --- | --- | --- | --- |
|  |  | Reverse | ACAACATGCCCTTTACACCA |
| *JAK2* | Exon 20 | Forward | CTTGAAAACTTGGTATTTCCATCC |
|  |  | Reverse | TATAGAATGCCTCTCCCTCTGGGC |
| *JAK2* | Exon 21 | Forward | GCAGAGTAAAACATTATTTCCACC |
|  |  | Reverse | ATGGTCTTAACCCTATATATTCCC |
| *CRLF2* | qRT-PCR | Forward | AATGCCAGCAAATACTCCAGGAC |
|  |  | Reverse | GGTAATAAACCATCCAGCGACTTG |
| *CRLF2* | F232C | Forward | AACGCCTCCCAAACCAAAG |
|  |  | Reverse | GCACGCTGGGAATGAGAAA |
| *P2RY8-CRLF2* fusion | 1st | Forward | CACGAACACCTTCTCAAGCA |
|  |  | Reverse | GTCCCATTCCTGATGGAGAAA |
|  | nest | Forward | TGTTACCTGGAGACCCTCTG |
|  |  | Reverse | GTTGAATCTGCTCCTCCTTG |

Table S3. Impact of other genetic alterations on outcome depending on *IKZF1* deletion

|  | *IKZF1* deletion (+) | | | *IKZF1* deletion (-) | | |
| --- | --- | --- | --- | --- | --- | --- |
|  | Relapse | Non- Relapse | P value | Relapse | Non- Relapse | P value |
| N | 7 | 12 |  | 20 | 163 |  |
| *CDKN2A/B* |  |  | 1.0/1.0 |  |  | 0.20/0.36 |
| deletion (%) | 42.9/42.9 | 50/41.7 |  | 55/45 | 61/52 |  |
| *PAX5* |  |  | 1.0 |  |  | 0.94 |
| deletion (%) | 57.1 | 50 |  | 30 | 26.3 |  |
| *ETV6* |  |  | 0.51 |  |  | 0.75 |
| deletion (%) | 0 | 16.7 |  | 30 | 23.9 |  |
| *RB1* |  |  | 1.0 |  |  | 0.67 |
| deletion (%) | 14.3 | 14.3 |  | 10 | 4.9 |  |
| *BTG1* |  |  | 1.0 |  |  | 0.46 |
| deletion (%) | 0 | 0 |  | 15 | 7.4 |  |
| *EBF1* |  |  | 0.52 |  |  | 0.74 |
| deletion (%) | 28.6 | 8.3 |  | 10 | 10.4 |  |
